# Mortality from non-communicable diseases and associated risk factors in Zambia; analysis of the sample vital registration with verbal autopsy 2015/2016

**DOI:** 10.1186/s12889-024-18150-4

**Published:** 2024-03-01

**Authors:** Emmanuel Musonda, Peter Mumba, Jacob R.S. Malungo

**Affiliations:** Department of Population Studies, School of Humanities and Social Sciences, University of Zambia, Lusaka, Zambia

**Keywords:** Verbal autopsy, NCDs, Risk factors, Africa, Zambia

## Abstract

**Background:**

Non-communicable diseases (NCDs) are the world’s growing cause of preventable illness, disability, morbidity, and mortality which account for 71% of deaths. The aim of this study was to determine the factors associated with mortality from NCDs among persons aged 15 years and above in Zambia.

**Methodology:**

The study used data from Sample Vital Registration with Verbal Autopsy (SAVVY) 2015/16 (Zambia). A total of 3529 Verbal Autopsy were completed in the study, with only 2599 of death where among people aged 15 years and above. Three-level data analysis was applied; univariate analysis, bivariate analysis, and multivariate analysis (binary logistic regression).

**Findings:**

The overall number of deaths from NCDs was 28.81%. Stratified analysis by gender showed that deaths from NCDs were higher among women (32.60%) as compared to men (26.25%). Among all persons, dying from NCDs was associated with tobacco use, age, and education. Tobacco use was negatively associated with mortality from NCDs (adjusted odds ratio [aOR] = 0.68; 95% confidence interval [CI]: 0.48–0.98). Age was positively associated with the odds of dying from NCDs among persons aged 45–59 years (aOR = 3.87, 95% CI: 2.13–7.01), 60–74 years (aOR = 12.05, 95% CI: 6.44–22.55), and 75 + years (aOR = 15.16, 95% CI: 7.93–28.97). The likelihood of dying from NCDs was higher among persons with secondary education as compared to those with no education (aOR = 1.93, 95% CI: 1.11–3.33).

**Conclusion:**

The findings from this study suggest that public health interventions targeting NCDs need to consider behavioural factors, especially tobacco use which exposes people to second-hand smoke. We also recommend large-scale national-level studies to further examine the contribution of each factor leading to mortality from NCDs.

**Supplementary Information:**

The online version contains supplementary material available at 10.1186/s12889-024-18150-4.

## Introduction

Non-communicable diseases (NCDs) have been seen to be the global disease burden due to the high probability of dying which accounts for 71% of deaths [1]. In most low and middle-income countries, the prevalent NCDs are coronary heart disease, hypertension, stroke, diabetes, asthma along with chronic hepatic and renal disease. The increasing burden of NCDs is potentially overwhelming to Africa’s inadequately financed health services [2]. The prevalence of mortality related to NCDs is often linked with the concept of epidemiological transition. This refers to a shift in patterns of mortality and disease during which infectious disease pandemics are gradually replaced by degenerative and human-made illnesses as the primary cause of death [3].

There are five well-known risk factors that are associated with NCDs: physical inactivity, insufficient intake of fruits and vegetables, being overweight or obese, smoking, and alcohol consumption [2, 4–7]. These factors can be classified into two main categories: lifestyle or behavioural, and biological factors. In Zambia, there has been a significant rise in the consumption of alcohol and tobacco, as well as an increase in the consumption of fats, sugar, and animal products in the year 2017. Additionally, there has been a decline in physical activity leading to a sedentary way of life that is linked with obesity, diabetes, and hypertension [8].. This increase may have been caused by changes in lifestyle, socioeconomic factors, or insufficient public health measures [9, 10].

Demographic and socioeconomic factors have been associated with mortality from NCDs. Advanced age, being male, urban residency, and lower education level have been associated with mortality from NCDs [11–13]. Being single is also a key risk factor for mortality from NCDs [14]. Occupational risk factors are significant indicators of mortality from NCDs. An individual’s job and work environment can contribute to the development or worsening of mortality from NCDs. For instance, people with lower-status occupations such as labourers and service workers have higher chances of dying due to high exposure to life-threatening events and injuries because the type of occupation varies in levels of exposure to hazards [15–18].

There is limited evidence about the risk factors associated with mortality from NCDs among persons aged 15 years and above in Zambia, using a nationally representative sample [19]. Therefore, this study investigated mortality from NCDs and associated risk factors in Zambia. The research findings will provide an opportunity to generate evidence that can be leveraged towards revising policies and interventions geared towards managing and averting fatalities due to NCDs across Zambia.

## Methods

### Data source

This study used data from Sample Vital Registration with Verbal Autopsy (SAVVY) 2015/16 [Zambia] to determine the factors associated with mortality from NCDs. The SAVVY (2015/16) was conducted by the Department of National Registration, Passport and Citizenship (DNRPC), in collaboration with the Central Statistical Office (CSO) and the Ministry of Health (MoH). The study was conducted in 10 provinces. The survey used the WHO standard methodology for 2015/16 SAVVY to report the leading causes of death in Zambia. The SAVVY was a cross-sectional retrospective and nationally representative survey. The main objective of the survey was to produce estimates of nationally representative age and sex cause-specific mortality fractions in Zambia (SAVVY, 2015/16) [20]. The SAVVY used three different types of WHO standardized data collection instruments: The Verbal Autopsy (VA) Questionnaire for deaths of children under the age of 4 weeks, the VA Questionnaire for deaths of children between the ages of 4 weeks to 14 years, and the VA Questionnaire for deaths of adults aged 15 and older. The following questions were used as the foundation for VA interviews: " Is there a usual member of this household who died in the last 12 months?“; “Was this person male or female?“, and “How old was this person?” The VA questionnaire for deaths of people aged 15 years and over, which was the questionnaire of interest to the study, then collected more detailed information about the deceased person on age at death, sex, marital status, occupation status at the time of death, education level, cause of death, and risk factors.

**Fig. 1:**
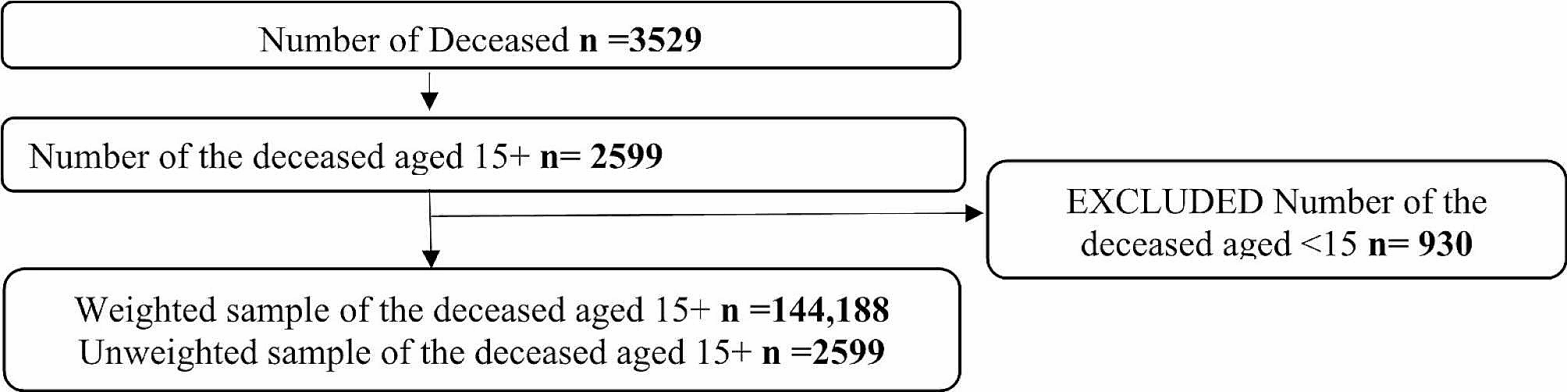
Sample selection and inclusion criteria

The analysis was based on the number of people aged 15 years and above whose cause of death was classified to be NCDs. A total of 3,529 deaths were reported. The study excluded those who were exactly 14 years and below and it only included those who were 15 years and above. The sample size for the study was 2599 (Unweighted) and 144,188 (Weighted) (Fig. 1). Figure 1 shows the sample selection and inclusion criteria.

### Outcome variable

The outcome variable was created by merging selected NCDs (Disease of the circulatory system, cancer, chronic respiratory condition, endocrine, nutritional and metabolic, disease of the nervous system and disease of the digestive system). The dependent variable was constructed as follows; A discrete binary variable coded as (0) if the immediate or underlying cause of death was not NCDs and (1) if the immediate or underlying cause of death was NCDs.

### Explanatory variables

The following are the variables that were used in the analysis of mortality from NCDs and associated risk factors in Zambia, which were chosen based on existing data and the availability of data. The variable age of the respondents was categorized as: 15–29 (treated as a reference category), 30–44, 45–59, 60–74, and 75+. The number of deaths from NCDs in this study was small, and for greater clarity of tabled data or easier identification pattern fifteen-year age group was used [21]. The education level variable had four categories: none, primary, secondary and tertiary education. In all the regression analyses respondents with no education were treated as a reference category. In terms of the residence variable, it was categorized as rural (treated as a reference category) and urban. The marital status variable was categorized as never married which was used as the reference category, married, widowed and separated/widow. Occupation was recoded as follows: skilled agriculture, managers/professionals, craft/ related trade works, market sales works, plant and machine operator, and elementary occupation. Region was recorded as Central, Copperbelt, Eastern, Luapula, Lusaka, Muchinga, Northern, North Western, Southern and Western.

In addition, with the expectation of age and sex, independent variables do not directly influence the dependent variable of mortality from NCDs. Hence, they operate through the identified intermediate variables which are poor diet, alcohol consumption, tobacco use and healthcare utilization. Age and sex have both direct and indirect influences on the outcome variable [22]. 2015/16 Zambia SAVVY did not collect any information on the deceased diet when they were alive. However, it did ask the question “Did the deceased have malnutrition?” Malnutrition is defined as deficiencies, excesses or imbalances in a person’s intake of energy and or nutrients [23]. In the interest of the study, the malnutrition variable was considered to be a proxy for poor diet and it was phrased as “Did the deceased have a poor diet?” Poor diet is defined as a serious condition that occurs when a person’s diet does not contain the right amount of nutrients. Lastly, the place of death (hospital or other places, home) was used also in the analysis as a proxy for access to health care during the terminal illness.

### Statistical analysis

The statistical analysis was done using STATA version 15. Three levels of analysis were employed, that is univariate, bivariate and multivariate analysis. Univariate analysis was performed to establish the percentage distribution of the deceased persons aged 15 years and above by the variables (socio-economic, demographic and behavioural risk factors). A stratified analysis by gender was used in the study at bivariate and multivariate analyses. Stratified analysis was performed because several studies have shown that there are significant differences in mortality rates between males and females. For example, males often have higher mortality rates than females for certain diseases and conditions, such as cardiovascular disease and some types of cancer [24–26].

Under bivariate analysis, the Chi-square test of independence was employed to establish the association between all the independent variables and mortality from NCDs, with row percentages applied to show the distribution of responses within each independent variable. Lastly, multivariate binary logistic regression was performed to determine the likelihood of dying from NCDs with each independent variable. Adjusted odds ratios (aOR) and their 95% confidence interval (CI) were presented. The level of significance used in the study was *p* = 0.05. In this study, behavioural risk factors are adjusted by age, sex, marital status, residence, region, education and occupation. The variance inflation factor (VIF) was employed to determine whether multi-collinearity existed among independent variables. There were no concerns with multi-collinearity in any of the variables (all VIF < 5) (Supplementary file Table 1). As a result of the survey’s sample design, sample weights were applied using the Stata svy command throughout the analysis to adjust for under- and overcounting. To make sure that our analysis appropriately represents the target population, sample weights were used. We attempted to improve the generalizability of our findings by adjusting for any discrepancies introduced during the sampling procedure by assigning weights to observations.

### Ethical considerations

The 2015/16 SAVVY survey was carried out with ethical approval from the University of Zambia Biomedical Research Ethics Committee. Given that VA involved interviewing the next of kin of the deceased, written informed consent was diligently obtained from each interviewee before proceeding with the interviews. All survey participants willingly volunteered to participate, and the dataset used, authorized by the Ministry of Home Affairs and Internal Security, was anonymized before being made publicly accessible.

## Results

### Response rate

During the baseline census, 4,350 deaths were reported; 3,529 of these deaths had verbal autopsies completed. There were 2,599 recorded deaths among the participants in the study who were 15 years and above. 

### Descriptive

Table 1 shows the distribution of the deceased persons aged 15 years and above who were sampled and considered for analysis of the factors associated with mortality from NCDs. The study showed that 29% of deaths occurred to persons aged 30–44. The majority of the deaths (60%) were recorded among males. More than half (57%) of the deaths were recorded among urban residents. About 44% of the deaths were recorded among those with only primary education and more than half of deceased persons (52%) were married. With regard to region, Lusaka had the highest number of death (20%) while North Western province had the lowest number of deaths (6%). Most of the deaths in terms of occupation were observed among the skilled farmers (49%).

Table 1 also presents the counts and per cent distribution of the risk behaviours and health factors. About 26% of deaths were due to tobacco use. Almost half (47%) of the deaths were due to alcohol consumption. Forty-six per cent of deaths were due to lack of health care access.

**Table 1:** Characteristics of the study population

Variables	Unweighted		Weighted	
	Number	Per cent	Number	Per cent
**Age**				
15–29	463	17.7	25,580	17.7
30–44	726	28.8	41,463	28.8
45–59	538	20.1	28,966	20.1
60–74	410	15.9	22,895	15.9
75+	462	17.5	25,282	17.5
**Sex**				
Male	1529	59.6	85,904	59.6
Female	1070	40.4	58,284	40.4
**Residence**				
Rural	1152	42.6	61,494	42.6
Urban	1447	57.4	82,695	57.4
**Education Attainment**				
No education	317	12.3	16,390	12.3
Primary	1087	44.3	59,105	44.3
Secondary	827	35.3	47,145	35.3
Higher	180	8.1	10,849	8.1
**Marital Status**				
Never Married	496	19.9	28,048	19.9
Married	1307	52.3	73,573	52.3
Divorced/Separated	315	11.9	16,740	11.9
Widow	418	15.9	22,359	15.9
**Region**				
Central	181	10.1	14,543	10.1
Copperbelt	445	14.6	21,084	14.6
Eastern	223	8.6	12,418	8.6
Luapula	264	9.2	13,376	9.3
Lusaka	454	20.2	28,148	20.2
Muchinga	196	8.5	12,220	8.5
Northern	199	8.6	12,465	8.6
North Western	181	5.7	8259	5.7
Southern	202	7.1	10,173	7.1
Western	254	7.3	10,503	7.3
**Occupation**				
Skilled Agriculture	885	49.4	46,754	49.4
Managers/Professionals	100	5.6	5980	5.6
Craft/ Related trade works	75	4.1	4154	4.1
Market sales works	104	5.8	5170	5.8
Plant& machine operator	271	15.1	15,594	15.1
Elementary occupation	358	19	18,176	19
**Tobacco use**				
Yes	612	26.4	34,584	26.4
No	1769	73.6	96,287	73.6
**Alcohol Consumption**				
Yes	1117	47	63,838	47
No	1341	53	72,042	53
**Poor Diet**				
Yes	43	1.5	2103	1.5
No	2463	98.5	136,465	98.5
**Health Care Access**				
Yes	1374	53.5	76,310	53.5
No	1196	46.5	66,295	46.5
Total	2599	100	144,188	100

Table 2 shows the association between mortality from NCDs and demographic and socio-economic factors among people in Zambia aged 15 years and above stratified by gender. The findings reveal that mortality from NCDs was associated with age, sex, educational attainment, marital status, region and occupation (*p* < 0.05) for all adults aged 15 years and above.

Further analysis demonstrated that males showed significant associations between NCDs-related deaths with age, education level, marital status and occupation (*p* < 0.05). On the other hand, among females, mortality from NCDs was significantly associated with age, marital status, and region (*p* < 0.05).

Table 2 further depicts the association between mortality from NCDs and behavioural and health factors. For all persons, mortality from NCDs was associated with tobacco use, alcohol consumption and poor diet (*p* < 0.05). Among males, mortality from NCDs was associated with tobacco use and alcohol consumption (*p* < 0.05). For females none of the behavioural and health factors were significantly associated with mortality from NCDs.

**Table 2:** Association between mortality from NCDs and risk factors stratified by gender among persons aged 15 years and above in Zambia

Variables	Mortality from NCDs							
	Male			Female			All	
	Number (% NCDs)	p		Number (% NCDs)	p		Number (% NCDs)	p
**Age**		**0.0000**			0.0000			0.0000
15–29	1163 (8.00)			1092 (9.90)			2255 (8.81)	
30–44	2280 (8.54)			2520 (17.07)			4800 (11.57)	
45–59	5002 (27.59)			3885 (35.86)			8887 (30.68)	
60–74	7628 (53.56)			4587 (53.02)			12,215 (53.35)	
75+	6474 (52.70)			6914 (52.95)			13,387 (52.95)	
**Residence**		0.1531			0.2302			0.0767
Rural	8554 (24.05)			7891 (30.43)			16,446 (26.74)	
Urban	13,993 (27.80)			11,106 (34.32)			25,100 (30.35)	
**Education Attainment**		0.0076			0.2416			0.0041
None	1799 (30.12)			3935 (37.78)			5734 (35.99)	
Primary	7223 (21.97)			7988 (30.45)			15,211 (25.773)	
Secondary	8908 (26.94)			3936 (27.95)			12,844 (27.24)	
Higher	3085 (37.19)			909 (35.59)			3994 (36.81)	
**Marital Status**		0.0000			0.0000			0.0000
Never Married	2217 (11.95)			1521 (16.03)			3739 (13.33)	
Married	16,287 (31.72)			7031 (31.62)			23,318 (31.69)	
Divorced/Separated	1388 (16.71)			1852 (21.96)			3241 (19.36)	
Widow	2355 (39.37)			8101 (49.47)			10,456 (46.76)	
**Region**		0.0938			0.0030			0.0008
Central	2074 (23.35)			1230 (21.73)			3304 (22.72)	
Copperbelt	3040 (21.45)			2790 (40.37)			5830 (27.65)	
Eastern	1750 (24.65)			1763 (33.16)			3515 (28.29)	
Luapula	1519 (18.89)			1086 (20.36)			2605 (19.47)	
Lusaka	5543 (33.00)			4551 (36.85)			10,093 (34.62)	
Muchinga	2163 (30.59)			2097 (40.73)			4261 (34.92)	
Northern	2373 (29.93)			1597 (35.19)			3969 (31.87)	
North Western	1209 (27.31)			956 (24.94)			2165 (26.21)	
Southern	1931 (29.16)			1632 (45.97)			3563 (35.02)	
Western	945 (26.25)			1295 (22.96)			2241 (21.34)	
**Occupation**		0.0000			0.2969			0.0001
Skilled Agriculture	7670 (28.24)			6397 (32.63)			14,067 (30.09)	
Managers/Professionals	2124 (50.76)			659 (36.70)			2783 (46.53)	
Craft/ Clerks	1524 (39.18)			65 (24.61)			1589 (38.25)	
Market sales works	456 (14.71)			928 (44.84)			1383 (26.76)	
Plant& machine operator	2518 (18.14)			1801 (23.93)			4319 (27.69)	
Elementary occupation	2725 (18.14)			902 (28.62)			3628 (19.96)	
**Tobacco use**		0.0001			0.3102			0.0004
Yes	5665 (19.67)			2197 (38.02)			7863 (22.73)	
No	15,070 (30.67)			15,226 (32.30)			30,297(31.46)	
**Alcohol Consumption**		0.0000			0.3504			0.0000
Yes	10,574 (21.13)			4092 (29.65)			14,666 (22.97)	
No	10,964 (35.22)			13,676 (33.43)			24,640 (34.20)	
**Poor Diet**		0.5820			0.1119			0.0133
Yes	80 (7.41)			183 (17.83)			263 (12.49)	
No	21,783 (26.8)			18,459 (33.40)			40,242 (29.49)	
**Health Care Access**		0.6291			0.5634			0.4636
Yes	12,285 (27.08)			10,374 (33.52)			22,659 (29.69)	
No	10,122 (73.51)			8565 (31.64)			18,687 (28.18)	
**Total**	22,548 (26.25)			18,998 (32.60)			41,545 (28.81)	

Source: SAVVY 2015/16, Note: These estimates are weighted

Table 3 shows the adjusted odds ratio for mortality from NCDs and selected explanatory variables stratified by gender. Among all persons aged 15 years and above, mortality from NCDs was associated with tobacco use, age and education. Persons that used tobacco were 32% less likely to die from NCDs when compared to those that did not use tobacco (aOR = 0.68; 95% CI: 0.48–0.98). Persons aged 45–59 years, 60–74 years and 75 + years were more likely to die from NCDs when compared to those aged 15–29 years (aOR = 3.87, 95% CI: 2.13–7.01), (aOR = 12.05, 95% CI: 6.44–22.55), and (aOR = 15.16, 95%CI: 7.93–28.97) respectively. The odds of dying from NCDs increased by 93% among persons with secondary education as compared to those with no education (aOR = 1.93, 95%CI: 1.11–3.33). Mortality from NCDs was not associated with alcohol consumption, poor diet, health care access, sex, residence, marital status, region and occupation.

In the male population, mortality from NCDs was associated with age, marital status and occupation. Males that used tobacco were 42% less likely to die from NCDs when compared to those who did not use tobacco (aOR = 0.58, 95% CI:0.37–0.88). The odds of dying from NCDs were positively associated with advanced ages 45–59 years, 60–74 years and 75 years and above (aOR = 4.27, 95% CI: 1.91–9.56), (aOR = 13.83, 95% CI: 6.11–31.32) and (aOR = 18.05, 95% CI: 7.56–43.11) respectively. Widowed men were 69% less likely to die from NCDs when compared to single men (aOR = 0.31, 95% CI: 0.11–0.85). Males who were managers or professionals had almost 3 times higher odds of dying from NCDs when compared to those with skilled agriculture type of occupation (aOR = 2.86, 95% CI: 1.26–6.52). Mortality from NCDs was not associated with alcohol consumption, poor diet, health care access, residence, education and region (Table 3).

However, among females, mortality from NCDs was only associated with age. Females who were aged 45–59 years, 60–74 years and 75 years and above had higher odds of dying from NCDs as compared to those aged 15–29 years (aOR = 3.41, 95% CI: 1.28–9.08), (aOR = 9.93, 95% CI: 3.52–28.04) and (aOR = 11.53, 95% CI: 4.07–32.64) respectively. Mortality from NCDs was not associated with tobacco use, alcohol consumption, poor diet, health care access, residence, education attainment, marital status, region and occupation (Table 3).

**Table 3:** Adjusted odds ratio for mortality from NCDs and selected explanatory variables

Variables	Male			Female			All	
	aOR	95% CI		aOR	95% CI		aOR	95% CI
**Tobacco use**								
Yes	0.58**	(0.37–0.88)		1.17	(0.57–2.43)		0.68**	(0.48-98)
No(RC)	1			1			1	
**Alcohol Consumption**								
Yes	0.7	(0.14–1.05)		0.95	(0.55–1.63)		0.77	(0.55–1.07)
No(RC)	1			1			1	
**Poor Diet**								
Yes	0.46	(0.05–3.97)		0.69	(0.21–2.29)		0.59	(0.19–1.77)
No(RC)	1			1			1	
**Health Care Access**								
Yes	1.19	(0.81–1.76)		1.34	(0.81–2.21)		1.27	(0.94–1.73
No	1			1			1	
**Age**								
15–29(RC)	1			1			1	
30–44	0.83	(0.37–1.84)		1.87	(0.71–4.85))		1.16	(0.64–2.11)
45–59	4.27***	(1.91–9.56)		3.41**	(1.28–9.08)		3.87***	(2.13–7.01)
60–74	13.83***	(6.11–31.32)		9.93***	(3.52–28.04)		12.05***	(6.44–22.55)
75+	18.05***	(7.56–43.11)		11.53***	(4.07–32.64)		15.16***	(7.93–28.97)
**Sex**								
Female(RC)							1	
Male							0.91	(0.63–1.29)
**Residence**								
Rural (RC)	1			1			1	
Urban	0.81	(0.49–1.35)		0.88	(0.49–1.62)		0.82	(0.56–1.21)
**Education Attainment**								
No education(RC)	1			1			1	
Primary	1.46	(0.69–3.12)		1.6	(0.83–3.08)		1.47	(0.90–2.41)
Secondary	1.86	(0.84–4.11)		2.18	(0.97–4.88)		1.93**	(1.11–3.33)
Higher	1.54	(0.57–4.17)		4.71	(0.65–34.46)		1.65	(0.74–3.70)
**Marital Status**								
Never Married(RC)	1			1			1	
Married	0.7	(0.34–1.43)		1.63	(0.62–4.26)		0.94	(0.54–1.61)
Divorced/Separated	0.79	(0.28–2.21)		0.92	(0.31–2.74)		0.67	(0.33–1.38)
Widow	0.31**	(0.11–0.85)		1.9	(0.64–5.56)		0.79	(0.40–1.56)
**Region**								
Central (RC)	1			1			1	
Copperbelt	1.4	(0.67–3.33)		1.4	(0.47–4.23)		1.24	(0.63–2.44)
Eastern	1.46	(0.60–3.56)		1.02	(0.33–3.20)		1.05	(0.51–2.17)
Luapula	1.02	(0.38–2.68)		0.93	0.29–2.94)		0.79	(0.37–1.69)
Lusaka	1.88	(0.83–4.21)		1.58	(0.53–4.73)		1.46	(0.74–2.87)
Muchinga	1.9	(0.77–4.71)		2.27	(0.73–7.03)		1.68	(0.81–3.47)
Northern	0.97	(0.41–2.31)		0.98	(0.31–3.11)		1.89	(0.43–1.82)
North Western	1.58	(0.59–4.27)		1.98	(0.69–5.66)		1.43	(0.69–2.97)
Southern	1.32	(0.54–3.26)		3.05	(0.94–9.90)		1.66	(0.80–3.44)
Western	1.13	(0.41–3.19)		1.68	0.55–5.08)		1.19	(0.56–2.56)
**Occupation**								
Skilled Agriculture(RC)	1			1			1	
Managers/Professionals	2.86***	(1.26–6.52)		0.51	(0.07–3.59)		1.96	(0.94–4.11)
Craft/ Clerks	1.66	(0.72–3.81)		2.33	(0.24–22.23)		1.65	(0.76–3.57)
Market sales works	0.69	(0.29–1.63)		1.63	(0.56–4.71)		1.03	(0.55–1.96)
Plant& machine operator	1.3	(0.66–2.55)		1.04	(0.49–2.17)		1.26	(0.77–2.05)
Elementary occupation	0.71	(0.40–1.25)		0.95	(0.33-70)		0.79	(0.48–1.32)

P-value in the parenthesis; *** *p* < 0.01, ** *p* < 0.05, * *p* < 0.1, RC: Reference Category, AOR: Adjusted Odds Ratio

The study findings reveal that, for both smokers and non-smokers, the likelihood of dying from NCDs-related deaths increases with age. Adult smokers aged between 45 and 59 were two times more likely to die from NCDs compared to those aged 15–29 (OR = 2.35, 95% CI 0.86–6.40). Similarly, for non-smoker adults aged 45–59, the risk was four times higher of dying from NCDs compared to adults aged 15–29 (OR = 4.83, 95% CI 3.09–7.54). Smokers aged 60–74 were six times more at risk of dying from NCDs compared to smokers aged 15–29 (OR = 6.94, 95% CI 2.66–18.11). For non-smokers, the risk was much higher for those aged 60–74 compared to those aged 15–29 (OR = 13.30, 95% CI 8.44–20.95). Both smokers and non-smokers aged 75 and over were 11 times more likely to die from NCDs compared to those aged 15–29 (OR = 11.36, 95% CI 4.44–29.05; OR = 11.25, 95% CI 7.21–17.55) (Table 4).

**Table 4:** Unadjusted odds ratio for mortality from NCDs and age stratified by smokers and non- smokers

Variables	Smokers			Non-smokers	
	UOR	95% CI		UOR	95% CI
**Age**					
15–29(RC)	1			1	
30–44	0.63	0.118–2.21		1.,55	0.97–2.49
45–59	2.35	0.86–6.40		4.83	3.09–7.54
60–74	6.94	2.66–18.11		13.3	8.44–20.95
75+	11.36	4.44–29.05		11.25	7.21–17.55

P-value in the parenthesis; *** *p* < 0.01, ** *p* < 0.05, * *p* < 0.1, RC: Reference Category, UOR: Unadjusted Odds Ratio

## Discussion

This study aimed to investigate the factors associated with mortality from Non-Communicable Diseases (NCDs) in Zambia. Among persons aged 15 years and above, NCDs accounted for 28.81% of overall deaths. These results are consistent with the 2018 WHO report, which stated that mortality in Zambia accounted for 29% [27]. The overall deaths from NCDs were higher among women at 32.60% compared to 26.25% among males. This contrast with another study that showed more male deaths from NCDs compared to female deaths [28]. This may be attributed to the fact that women tend to experience higher rates of obesity, especially after childbirth, in comparison to men. This increased prevalence of obesity among women can place them at a greater risk of developing complications related to hypertension and other cardiovascular diseases. Pregnancy and postpartum periods can lead to weight gain, hormonal changes, and lifestyle adjustments that, when combined with pre-existing factors, can contribute to a higher risk to cardiovascular diseases and hypertension [29–31].

The study results indicate that tobacco use, age and education were factors associated with mortality from NCDs. Among males, mortality from NCDs was linked to smoking, age, marital status, and occupation. In contrast, among females, age was the sole factor associated with mortality from NCDs.

Males and all individuals who used tobacco had a lower likelihood of dying from NCDs compared to non-users. This association was stronger among all individuals and males but not among females. This finding contradicts other studies that have shown smoking to increase the risk of mortality [32–34]. One possible reason that could explain our findings is that smoking, as a single behavioral risk factor, cannot be monitored independently of other contributing factors to mortality from NCDs. For example, some individuals who do not smoke may still be exposed to other risk factors such as obesity, poor diet, and lack of physical activity, which can increase their chances of dying from NCDs [35].

As expected, advanced ages 45–59 years, 60–74 years and 75 years and above were associated with dying from NCDs among males alone, females alone and all persons. The results of this study are similar to two studies conducted in Zambia which indicated deaths from NCDs increased with an increase in age [36, 37]. This is because older age groups are associated with low physical activity as the result of physical disability which increases the chances of dying from NCDs [38].

Unexpectedly the findings from this study show that all deceased persons whose highest level of education was secondary education, had higher odds of dying from NCDs than those with no education at all. The finding from this study are in conflict with the finding of several studies which have consistently shown that higher education attainment is a strong indicator of social determinants of good health [39–41]. One possible reason could be that social desirability bias may have affected data reporting in a way that obscures the true association between education level and mortality from NCDs, which could serve as one explanation for the results [42, 43]. A possible explanation for this is variations is the extent to which one consumes alcohol, uses tobacco, exercises and eats nutritious foods [6, 44, 45]. The other reason is that persons with better socioeconomic status are associated with low physical activity and lifestyles that are more sedentary. In addition, higher incomes are also associated with affluent lifestyles including dietary modifications and obesity which are risk factors for dying from NCDs [46–48]. This association was only strong among all persons and not among females alone or males alone.

Our study demonstrated that being widowed was associated with lower odds of dying from NCDs as compared to single persons among males alone. This is similar to the finding of [14], who stated that a widowed person has a reduced risk of dying from NCDs as compared to single person. This could be because, in the Zambian culture set up, men who are widowed do not take long to get another wife. As a result, this lowers the chances of being associated with risky lifestyles such as poor diet, excessive alcohol consumption and tobacco use which are the risk factors of NCDs [7, 49].

Occupation is one of the important factors which incorporates the type of job and income one has. The findings from our study show that persons in managerial positions had a higher likelihood of dying from NCDs when compared to those who were in agriculture among males. This study finding is not consistent with studies conducted in Korea and South Africa [16, 50]. Both studies showed that people in lower occupation categories were more likely to experience adult mortality due to NCDs. It is conceivable that persons with high occupational status are usually associated with non-physically taxing activities and dietary modification which are risk factors for dying from NCDs. For instance, people with higher occupation status have adapted to the modern forms of transportation including vehicles and motorbikes which have contributed to being physically inactive. As a result, physical inactivity has been associated with obesity which is the risk factor for mortality from NCDs [33, 51]. The association was only strong among males alone and not among females alone and all persons.

### Limitation of study

Some limitations were noted in the study, firstly, the data used in the study is cross-sectional, meaning that causality cannot be established, and the time variation of events cannot be determined. Secondly, the accuracy and reliability of the verbal autopsy data is dependent on the quality of information provided by close relations of the deceased and the expertise of the interviewers, which can lead to recall bias and misclassifications when determining the cause of death. However, quality control measures were put in place during data collection, and qualified medical personnel and nosologists were used to establish and classify the causes of death. Thirdly, the low number of deaths overall did not allow for further analysis as it may have resulted in small sample sizes in some cells.

## Conclusion

The study indicates that socio-economic (such as education), behavioural risk factor (such as tobacco us) and some demographic (such as age) characteristics were associated with mortality from NCDs among all persons. Age was found to be one of the determinants of mortality from NCDs because the odds of dying from NCDs increased with a corresponding increase in age. Education was one of the factors that influenced mortality from NCDs, but unexpectedly, people with secondary education had higher odds of dying from NCDs than those with no education. All persons that used tobacco were less likely to die from NCDs. Amongst males, only tobacco use was negatively associated with dying from NCDs, whilst age and occupation was positively associated NCDs. Among females, only age was positively associated with mortality from NCDs. In this study, single risk factors do not attribute to mortality from NCDs. For instance, smokers had lower odds of dying from NCDs among males and all persons. Therefore, further research should be conducted to understand the influence of other risk factors (such as obesity, physical activity) on mortality from NCDs. In addition, as recommended in other studies, mortality from NCDs requires consistent research studies so that the factors leading to mortality from NCDs can be clearly understood. Therefore, governments and academia should invest more in the longitudinal type of research on examining the contributions of each factor leading to mortality from NCDs.

### Electronic supplementary material

Below is the link to the electronic supplementary material.

Supplementary Material 1

## Data Availability

The datasets produced and/or examined in the course of this study are not publicly accessible owing to their sensitive nature. Nevertheless, interested parties may request access to these datasets from the corresponding author, subject to reasonable and appropriate requests.

## Abbreviations

DNRPC: Department of National Registration Passport and Citizen
MoH: Ministry of Health
NCDs: Non-Communicable Diseases
SSA: Sub-Saharan Africa
SAVVY: Sample Vital Registration with Verbal Autopsy
VA: Verbal Autopsy
WHO: World Health Organization
