# Supplementary material for: Mortality from non-communicable diseases and associated risk factors in Zambia; analysis of the sample vital registration with verbal autopsy 2015/2016
Source: BMC Public Health. 2024 Mar 1;24:666. doi: 10.1186/s12889-024-18150-4 (PMC10908156; doi:10.1186/s12889-024-18150-4)
Supplement: Supplementary file 1 — Supplementary Material 1 [file 12889_2024_18150_MOESM1_ESM.docx]

**Supplementary file Table 1: Results of Multicollinearity Test**

| Variable | VIF |
| --- | --- |
| Tobacco use | 1.32 |
| Alcohol consumption | 1.35 |
| Poor diet | 1.01 |
| Healthcare access | 1.11 |
| Age group | 1.28 |
| Sex | 1.4 |
| Residence | 1.46 |
| Education attainment | 1.24 |
| Marital status | 1.35 |
| Region | 1.03 |
| Occupation | 1.44 |
| Mean VIF | 1.27 |
